# The Role of Working Memory in Dual-Target Visual Search

**DOI:** 10.3389/fpsyg.2019.01673

**Published:** 2019-07-31

**Authors:** Elena S. Gorbunova, Kirill S. Kozlov, Sofia Tkhan Tin Le, Ivan M. Makarov

**Affiliations:** School of Psychology, National Research University Higher School of Economics, Moscow, Russia

**Keywords:** visual attention, visual search, multiple targets, working memory, subsequent search misses

## Abstract

Visual search (VS) for multiple targets is especially error prone. One of these errors is called subsequent search misses (SSM) and represents a decrease in accuracy at detecting a second target after a first target has been found. One of the possible explanations of SSM errors is working memory (WM) resource depletion. Three experiments investigated the role of WM in SSM errors using a dual task paradigm. The first experiment investigated the role of object WM using a classical color change detection task. In the second and the third experiments, a modified change detection task was applied, using shape as the relevant feature. The results of our study revealed no effect of additional WM task on second target detection in dual-target VS. To this end, SSM errors are not related to WM resource depletion. On the contrary, WM task performance was violated by dual-target VS as compared to single-target VS, when the targets in VS task were defined by the same feature used in the WM task.

## Introduction

Visual search (VS) is a process of searching for targets among distracters. This task is very important in everyday life, as well as for some jobs (e.g., radiology, baggage screening). Nevertheless, VS is error prone. One of these errors is called subsequent search misses (SSM) and is observed in dual-target VS (e.g., Adamo et al., 2013). SSM is the decrease in accuracy at detecting a second target after a first target has been found.

The nature of SSM is as of yet unspecified. The first explanation of this phenomenon was proposed in radiological studies that supposed the second target omission to be related to a premature ending of the search. After finding the first target, the subject becomes “satisfied” with this result and does not search for any other possible targets (Tuddenham, 1962). Therefore, this phenomenon has been called satisfaction of search. However, searchers do continue searching after the first target is found (e.g., Fleck et al., 2010), which means that the “satisfaction” is not the only reason for SSM.

Alternative theories suggest that target similarity and resource depletion may play a role. According to perceptual set theory, the first-found target creates a perceptual bias, so the subject is more likely to find perceptually similar targets and less likely to find the targets that are perceptually dissimilar. Recent experiments (Gorbunova, 2017) provided some support for this theory as the SSM effect decreased with an increase in the number of shared features in two targets. Moreover, the SSM effect depends not only on the perceptual, but also on the conceptual target similarity (Biggs et al., 2015). The idea of perceptual bias is also consistent with the prevalence effect in VS: low prevalence reduces the probability of detecting targets, so the subjects are likely to miss the targets that are rare (Wolfe et al., 2005). The possible underlying mechanisms of the perceptual set can refer to perceptual priming or guidance. Still, the nature of how exactly this perceptual bias works is not completely clear. One of the possible mechanisms involves the role of working memory (WM) which is used to store target representations. This brings us to the third possible explanation of SSM errors – resource depletion (Cain and Mitroff, 2013).

The resource depletion account suggests that cognitive resources – attention and/or WM – are consumed by the first-found target. The constructs of attention and WM can sometimes describe overlapping concepts (e.g., Chun et al., 2011). Sometimes attention is understood as the control mechanisms of WM that selectively encode and maintain information in VWM. This idea is supported by the results of Schmidt et al. (2002) experiments where attentional cuing to a particular location can influence which objects are encoded into VWM. However, a recent experiment conducted by Tas et al. (2016) challenges this idea. During the retention interval, the irrelevant object appeared, and the participants were instructed to make overt and covert shifts of attention to it. Saccades to the secondary object produced interference with WM performance, but the covert shifts of attention did not produce the interference. The possible explanation may assume that the relationship between attention and WM is strongly dependent on the memorial demands of the orienting behavior.

Moreover, it has been shown that not all items encoded in visual WM are capable of affecting VS performance. For example, Olivers et al. (2006) experiments revealed that the presence of singleton distracters interfered more strongly with a VS task when it was accompanied by an additional memory task. This effect was not present or present in reverse when the slight modification of the paradigm was used (Downing and Dodds, 2004; Woodman and Luck, 2007). Later experiments revealed that this effect is dependent from the form of mapping and stimulus energy (Olivers, 2009). As not all memorized items influence the deployment of attention, the idea of two different kinds of WM representations was proposed: active memory items, which are stored visual WM and directly affect perception, and passive memory representation, which are also stored in visual WM system, but in a dormant state, and have a minor influence on visual selection (Olivers et al., 2011). This idea is supported by the results of recent experiments which revealed that immediately task relevant colors recruit attention to matching stimuli, whereas not immediately task relevant colors do not to interact with perceptual selection (Hollingworth and Hwang, 2013).

The SSM-errors explanations assume both attention and WM related mechanisms. The empirical support implies that attention has a strong contribution to SSM errors. Finding a first target increased the attentional effects of clutter on second target accuracy (Adamo et al., 2015). Individual differences studies revealed that second-target misses related to worse attentional modulation and vigilance (Adamo et al., 2017). A WM account suggests that the first-found target identities and/or locations are stored in WM at the time of the subsequent search which leaves few resources to find a second target. The search for the second target is different from the search for the first target, as the first-found target is still present on the search array, and acts as some form of a distracter, misdirecting the attention and/or diverting the resources from the subsequent search (Cain and Mitroff, 2013). Cain et al. (2014) provided support for this account. Dividing one multiple-target search into several single-target searches, separated by unrelated trials, effectively freed WM resources and eliminated SSM errors. Moreover, removing already found targets from the display or making them salient and easily segregated color singletons improved subsequent search accuracy (Cain and Mitroff, 2013).

There is a debate in visual WM literature over whether capacity is best defined as a resource. This resource is discussed as continuous and variable (van den Berg et al., 2012), or assumes the set of discrete, fixed-resolution representations (Zhang and Luck, 2008), or is related to interference between representations in WM (Oberauer and Lin, 2017). However, the resource depletion in SSM errors could be related to a continuous mnemonic resource being consumed, or because slots are used up, or perhaps because of interference.

The resource depletion in SSM errors could be because a mnemonic resource is being used up (a la continuous resource – van den Berg et al., 2012 PNAS style) or because slots are used up (Zhang and Luck, 2008, Nature), or perhaps because of interference (Oberauer and Lin, 2017, Psych Review).

Overall, both the perceptual bias and the resource depletion accounts predict that WM plays a key role in the SSM effect. Nevertheless, the exact kind of WM resources which cause resource depletion are not yet defined. They could be target identities (object WM) or the explored spatial locations (spatial WM). Experiments on standard single-target VS tasks revealed the role of both object and spatial WM. Woodman et al. (2007) investigated the role of object WM in VS. The participants performed a VS task during the delay interval of a visual WM task, a standard change-detection task, and separately. The two tasks were found to interfere with each other (interference for the VS task was measured by slopes sizes) when the search targets changed from trial-to-trial, which implies the target’s representations were encoded in visual WM during the VS. These results are relevant to the idea of the “attentional template” – the target representation which is stored in visual WM and used to guide attention during VS (Carlisle et al., 2011).

Stroud et al. (2012) revealed that a simultaneous search for two colors produced a dual-target cost, modulated by targets similarity (“split-target cost”) – as the similarity between the target colors decreased, search efficiency suffered. To this end, when two targets are dissimilar, they are apparently encoded as separate and discrete representations. In the latter experiments, participants searched for a target of a specific color while holding a color or a non-color item in WM (Menneer et al., 2019). Holding a color in WM caused the general disruption in attentional guidance to a color target, similar to that observed in dual-target search. Moreover, specific WM-color attracted fixations were observed, that is the evidence of colors in WM competing for attention.

Woodman and Luck (2004), as well as Oh and Kim (2004) experiments involved the comparison of a VS task performed during the retention interval of a spatial WM task, and a VS task tested in isolation. The spatial WM task included a location change detection task, in which the subjects had to memorize the locations of two sequentially presented dots. After the retention interval two dots were displayed simultaneously and the participants had to give a response to indicate whether a location change was detected. VS efficiency was impaired when the search and the memory tasks were performed concurrently, as compared with when the search task was performed separately.

However, Horowitz and Wolfe (1998) found no change in efficiency of VS when the search scene was continually shuffled while the observer was trying to search through it. Moreover, the results from a multiple-target paradigm, when the number of targets in the display was varied and the subjects were asked to report whether or not there were at least n targets present, revealed the reaction time (RT) as an accelerating function of n, which assumes memory-free search (Horowitz and Wolfe, 2001).

Thus, there are two potential candidates for WM resources falling under resource depletion thereby causing the omission of the second target in a dual-target VS: target identities (object WM) and observed locations (spatial WM). Based on the data from perceptual and conceptual target similarity, object WM representations seem more likely (Biggs et al., 2015; Gorbunova, 2017).

In Experiment 1 we used a color change-detection task similar to Woodman et al. (2007). In Experiment 2 a modified change detection task with shape features was used. Experiment 3 also assumed a change-detection task with different number of shapes for memorization. In all experiments, three conditions were used: a single VS task, a single WM task and a combined VS and WM task. If the dual-target VS and the WM task require the same resources, two kinds of interference are expected: first, the search in the dual-target condition would be worsened by the additional WM task, and second, the response accuracy in the WM task would worsen with an additional dual-target VS task.

## Experiment 1

### Materials and Methods

#### Participants

30 volunteers, 3 male, and 27 female, students of National Research University Higher School of Economics participated in the study. All of the participants were native Russian speakers with normal or corrected to normal vision. The age varied between 17 and 22 years (*M* = 18.93, *SD* = 1.14). All participants were naive to the experimental hypothesis.

The experiment included three conditions: a WM task, a VS task and a combined task for working memory and visual search (VS + WM). The order of presentation was counterbalanced across subjects. Articulatory suppression was used during the whole experiment to avoid the possibility of verbal coding.

#### Apparatus

Participants sat in a dark room 45 cm from a 19 in. LACIE electron 19 blue III monitor (screen resolution 1024 × 768, refresh rate 85 Hz). Stimuli were displayed with Psychopy v. 1.82.01, OS Ubuntu. Participant answers were registered with a standard keyboard and mouse.

### Working Memory Task

#### Stimuli

The stimuli were squares of highly discriminable colors: white, black, red, green, blue, and yellow. On each trial, four squares were displayed arranged around a fixation cross at the top, bottom, left and right. The stimuli size was 1.15° × 1.15°. The stimuli were presented on a gray background (CIE *xy* = 0.273, 0.304; luminance = 40.897 cd/m^2^) and the colors of the stimuli were varied each trial. There were always four items per display.

#### Procedure

At the beginning of the trial, a sample array with four colored squares was displayed for 500 ms. This was followed by 4000 ms ISI. After that, the test array appeared. The time limit for test array was 2000 ms, after that the test array was replaced with the sign “?,” appearing at the center of the screen. The participant’s task was to remember the initial colors of the squares of the sample array and to report if the test array is the same as the sample array or not. The response was given with two predefined buttons (“N” and “Z”) on the keyboard. The participant pressed the “space” bar on the keyboard to begin the next trial. The participant could take the small breaks during the experiment. The breaks were available at any moment, the time was unlimited. The participants stayed at the lab at the moment of the break, in order to eliminate the influence of the external environment. All participants took the breaks, usually 2–3 breaks during the session. The breaks were no longer than 2 min. The participants were instructed to perform both fast and accurately.

The condition consisted of 100 trials. On 50% of trials the test array was identical to the sample array, and on the other 50% the color of one randomly selected square was replaced by a color that was not present in the sample array.

A training session of five trials preceded the experiment.

### Visual Search Task

#### Stimuli

The stimuli were rectangles with gaps which could be at the top, bottom, right, or left. The stimuli size was 1.38° × 0.93°. According to previous SSM research paradigms (e.g., Fleck et al., 2010; Adamo et al., 2013), stimuli were designed to have different levels of salience by increasing their brightness: high (CIE *xy* = 0.272, 0.297; luminance = 14.155 cd/m^2^), medium (CIE *xy* = 0.272, 0.301; luminance = 21.653 cd/m^2^), and low (CIE *xy* = 0.272, 0.303; luminance = 28.475 cd/m^2^). On each trial, there were around 33% stimuli of each type. A target cue was displayed at the beginning of the trial and had black color (CIE *xy* = 0.267, 0.262; luminance = 1.073 cd/m^2^). The stimuli were presented on gray background (CIE *xy* = 0.273, 0.304; luminance = 40.897 cd/m^2^). There were always 20 items per display. On each trial there were one, two, or no targets present. For one target, it could be high-salient or low-salient, for two targets, one was always high-salient, and the other was always low-salient.

The stimuli were displayed at the corners of the screen (upper left and lower right on the 50% of the trials and upper right and lower left on the 50% of the trials) in order not to infer with the WM task stimuli. In the dual-target condition, the targets could appear in different zones in 50% of trials and in the same zone in 50% of trials.

**FIGURE 1:**
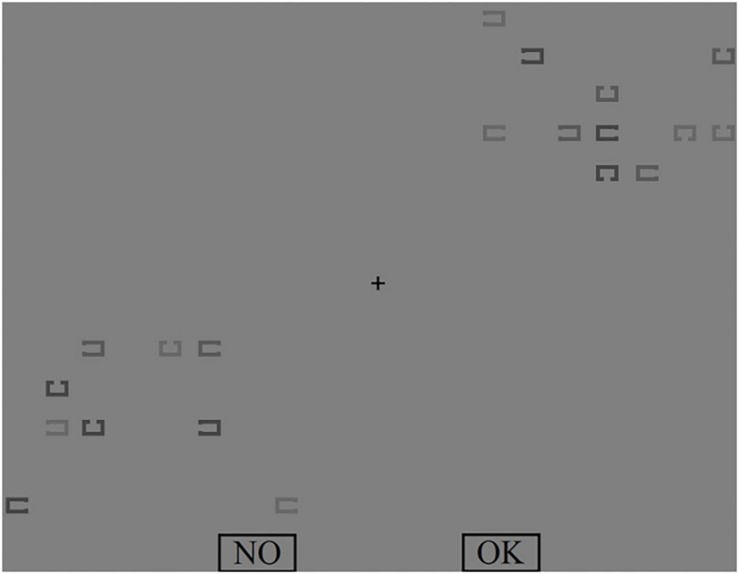
An example of visual search (VS) display.

There were “NO” and “OK” buttons at the bottom of the screen, size each 6.43° × 3.25°. These buttons were used for participant answers. An example of VS display is presented in Figure 1.

#### Procedure

The experiment consisted of 160 trials. In 40 trials the target was not present (catch-trials), 80 trials included one target (40 trials with a high-salient target and 40 trials with a low-salient target), 40 trials included two targets. The order of presentation was randomized.

The participant’s task was to find all the target stimuli or report their absence. The type of target stimuli (the gap location) was indicated at the beginning of each trial at the center of the screen using a black image of the target stimuli. This image was displayed for 1000 ms.

The participants reported the target stimuli by clicking them with the mouse. The participant reported the absence of target stimuli by clicking the “NO” button at the bottom of the screen. The participant made two clicks in each trial. For two targets, one click on each target was made. For one target, the first click was on target and the second on “OK” button. For no targets, two clicks on “NO” button were made. After the first target was found, it was still present on the screen.

Each trial had a limit of 20 s., after which the screen cleared. The participant pressed the “space” bar to begin the next trial. The participant could take the small breaks during the experiment. The participants were instructed to perform both fast and accurately.

A training session of five trials preceded the experiment.

### Visual Search + Working Memory Task

#### Stimuli

The stimuli were the same as the WM and VS tasks.

#### Procedure

**FIGURE 2:**
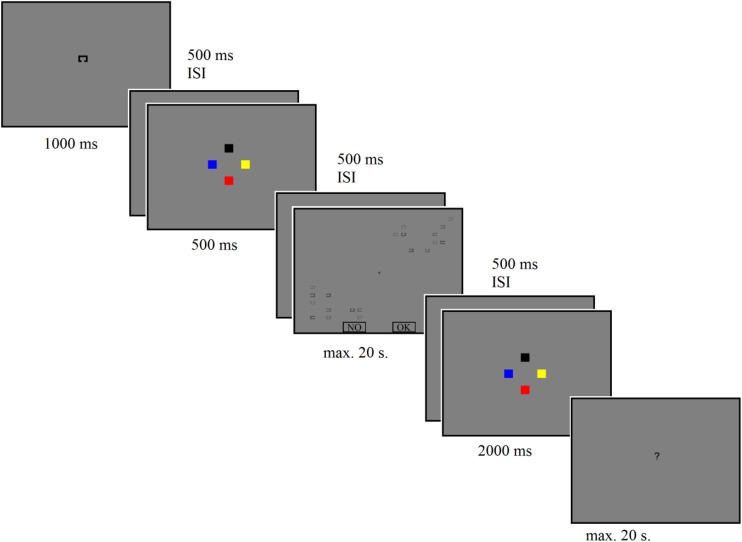
Example of the stimulus sequence on a single trial for combined condition on experiment 1. For better picture quality, the relative sizes of memory task squares, target at the beginning of the trial, and the “?” sign are two times bigger.

The trial started with the target presentation (1000 ms), followed by a 500 ms ISI. After that, the sample display with the WM task was displayed for 500 ms, followed by a 500 ms ISI. Then the search array was displayed. After the participant finished searching for targets (after two mouse clicks), a 500 ms ISI appeared, and the participant gave the answer to the memory task. An example of experimental trial is presented in Figure 2.

This condition consisted of 160 trials. In 40 trials the target was not present (20 trials without changing the color of the squares, 20 trials changing the color of the squares), 80 trials included one target [40 trials with high-salient target (20 trials without changing the color of the squares, 20 trials changing the color of the squares) and 40 trials with low-salient target (20 trials without changing the color of the squares, 20 trials changing the color of the squares)], other trials included two targets (20 trials without changing the color of the squares, 20 trials changing the color of the squares). The order of presentation was randomized.

A training session of five trials preceded the experiment.

### Results

For the VS, accuracy^1^ and RT for conditions with two targets and one low-salient target were compared to the single VS task and to the combined task. For the combined task, the analysis was conducted only for the correctly answered WM task trials.^2^ The accuracy analysis calculated the percentage of correct answers for the second low-salient target if the first high-salient target was found. RT was calculated separately for the first and for the second mouse click. RT was calculated only for correct trials. RTs higher and lower than 2 SD’s away from the mean for each participant were excluded from the analysis. Detailed results are presented in Table 1.

**TABLE 1:** The results of Experiment 1.

**Task**	**Condition**	**Index**	**Mean**	**SD**
WM alone task	WM alone	WM accuracy	84.83333	10.47849
VS+WM task	WM+VS, no targets	WM accuracy	75.5	12.35956
	WM+VS, 1 high salient target	WM accuracy	74.91667	14.16472
	WM+VS, 1 low salient target	WM accuracy	76.33333	15.06785
	WM+VS, 2 targets	WM accuracy	76.91667	15.0385
VS alone task	VS alone, no targets	VS accuracy	92.08333	7.134283
	VS alone, 1 high salient target	VS accuracy	70.75	16.50692
	VS alone, 1 low salient target	VS accuracy	65.25	19.76533
	VS alone, 2 targets	VS accuracy	49.55986	22.73461
VS+WM task	VS+WM, no targets	VS accuracy	81.21532	15.49733
	VS+WM, 1 high salient target	VS accuracy	68.03649	19.51811
	VS+WM, 1 low salient target	VS accuracy	61.44893	23.58185
	VS+WM, 2 targets	VS accuracy	49.16633	27.96119
VS alone task	VS alone, no targets	1^*st*^ click RT	5997.324	1019.468
	VS alone, 1 high salient target	1^*st*^ click RT	4289.653	621.4847
	VS alone, 1 low salient target	1^*st*^ click RT	4483.227	541.9617
	VS alone, 2 targets	1^*st*^ click RT	3710.432	507.5245
VS+WM task	VS+WM, no targets	1^*st*^ click RT	5759.598	1604.003
	VS+WM, 1 high salient target	1^*st*^ click RT	4409.7	1389.292
	VS+WM, 1 low salient target	1^*st*^ click RT	4508.816	1423.27
	VS+WM, 2 targets	1^*st*^ click RT	3991.881	1213.851
VS alone task	VS alone, no targets	2^*nd*^ click RT	238.8392	102.7953
	VS alone, 1 high salient target	2^*nd*^ click RT	2048.843	777.2456
	VS alone, 1 low salient target	2^*nd*^ click RT	1972.681	863.3099
	VS alone, 2 targets	2^*nd*^ click RT	1736.784	559.3306
VS+WM task	VS+WM, no targets	2^*nd*^ click RT	341.2473	291.4473
	VS+WM, 1 high salient target	2^*nd*^ click RT	1867.157	751.7514
	VS+WM, 1 low salient target	2^*nd*^ click RT	1934.086	770.3868
	VS+WM, 2 targets	2^*nd*^ click RT	1553.204	472.4248

For WM, accuracy was compared to the single WM task and for the combined task (for the one low-salient target and for the dual-target condition).

Data analysis was performed using SPSS 20.0. Repeated measures analyses of variance (rmANOVA) was used. Greenhouse-Geisser corrections were applied for significant Mauchly’s sphericity tests. For VS, the factors included the WM load (the VS task compared to the VS + WM task) and the number of targets (the one low-salient target condition compared to the dual-target condition). For WM, the factor was the additional VS task (the WM compared to the combined condition with one low-salient target and two targets). Pairwise comparisons (with Bonferroni adjustment) were used.

### Visual Search

#### Accuracy

**FIGURE 3:**
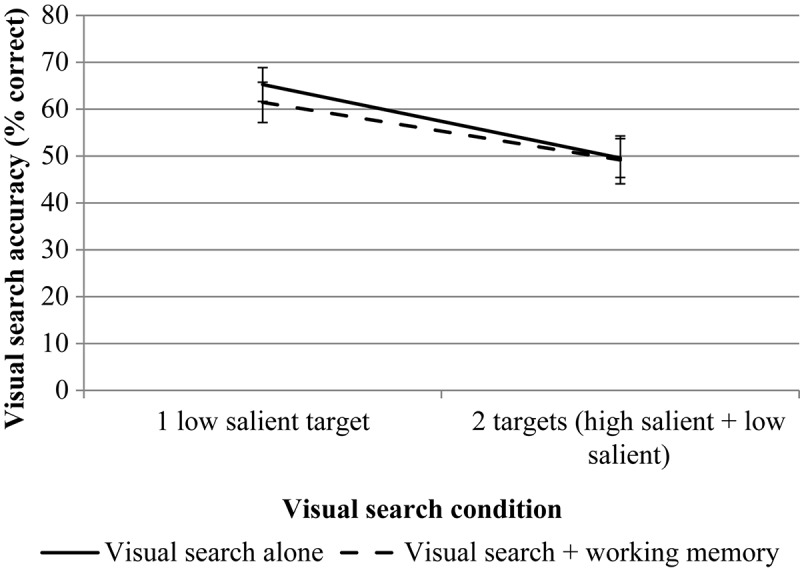
The results of experiment 1 (accuracy data for VS). Error bars represent standard error means.

RmANOVA revealed a significant effect for the number of targets, *F*(1, 29) = 26.94, *p* < 0.001, η*p*^2^ = 0.482. The effect of the WM load is not significant, *F*(1, 29) = 0.38, *p* = 0.54, η*p*^2^ = 0.013. The interaction is not significant, *F*(1, 29) = 0.78, *p* = 0.38, η*p*^2^ = 0.026. The results are presented in Figure 3.

#### Reaction Time

For the first mouse click, rmANOVA revealed a significant effect for the number of targets, *F*(1, 24^3^) = 97.99, *p* = 0.000, η*p*^2^ = 0.803. The effect of the WM load is not significant, *F*(1, 24) = 0.08, *p* = 0.787, η*p*^2^ = 0.003. The interaction is significant, *F*(1, 24) = 7.06, *p* = 0.014, η*p*^2^ = 0.227. Pairwise comparisons revealed no significant differences between different levels of load (the VS task compared to the VS + WM task) both for single low salient target condition, *p* = 0.709 and for dual target condition, *p* = 0.366.

For the second mouse click, rmANOVA revealed a significant effect for the number of targets, *F*(1, 25) = 9.76, *p* = 0.004, η*p*^2^ = 0.281. The effect of the WM load is not significant, *F*(1, 25) = 0.30, *p* = 0.590, η*p*^2^ = 0.012. The interaction is significant, *F*(1, 25) = 5.22, *p* = 0.031, η*p*^2^ = 0.173. Pairwise comparisons revealed no significant differences between different levels of load (the VS task compared to the VS + WM task) both for single low-salient target condition, *p* = 0.720 and for the dual-target condition, *p* = 0.897.

**FIGURE 4:**
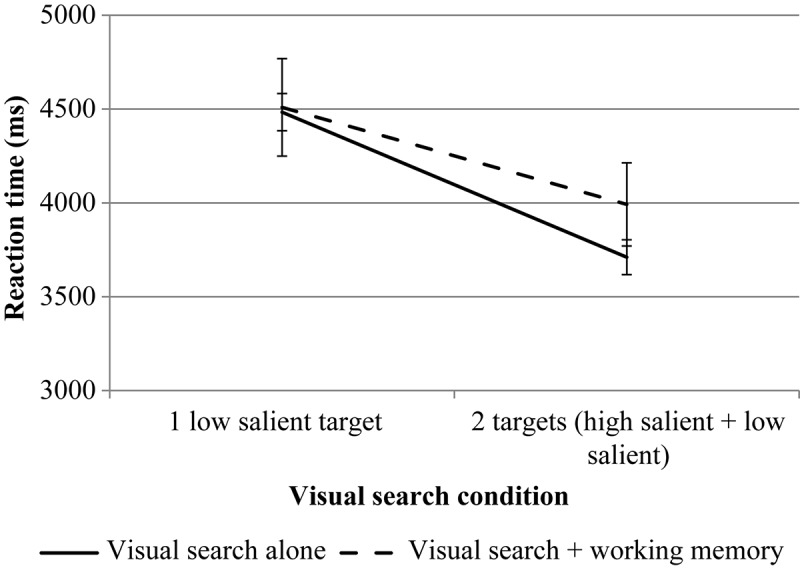
The results of experiment 1 (RT of the first mouse click for VS). Error bars represent standard error means.

**FIGURE 5:**
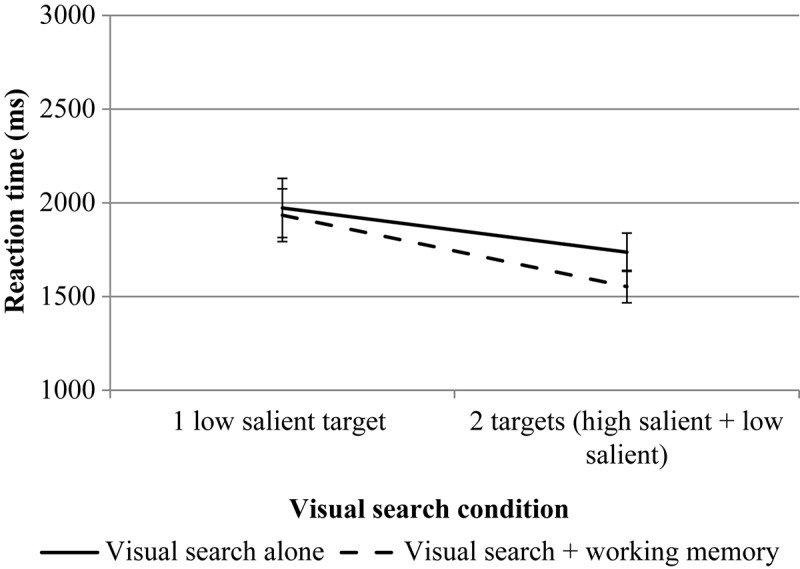
The results of experiment 1 (RT of the second mouse click for VS). Error bars represent standard error means.

The results are presented in Figures 4, 5.

#### Working Memory

**FIGURE 6:**
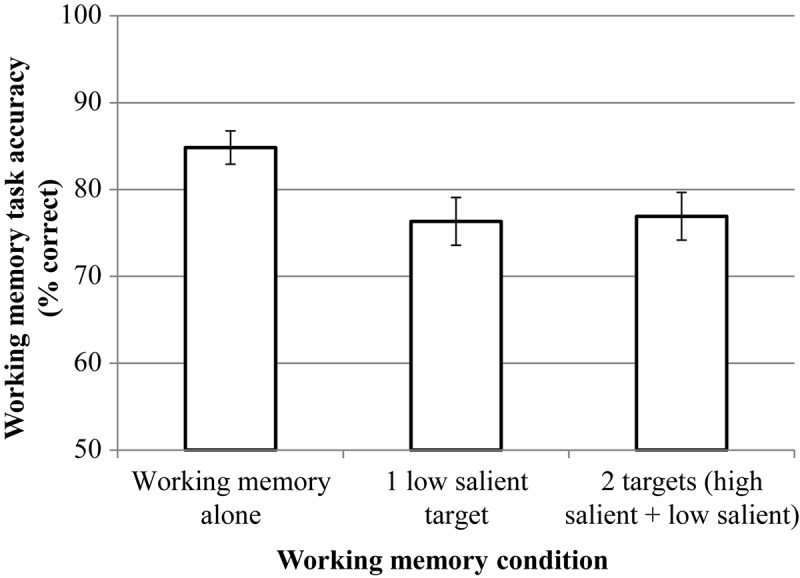
The results of experiment 1 (working memory (WM) task). Error bars represent standard error means.

RmANOVA revealed the significant effect of condition, *F*(2, 46) = 8.51, *p* = 0.002, η*p*^2^ = 0.227. But pairwise comparisons (Bonferroni corrected) did not reveal significant differences between the dual-target condition (*M* = 76.92, *SD* = 15.04) and the single low-salient target condition (*M* = 76.33, *SD* = 15,07), *p* = 0.721.^4^ The results are presented in Figure 6.

### Discussion

Our results revealed a significant effect for the number of targets: the SSM effect (the decrease in accuracy in the detection of a second, low-salient, target after the first, high-salient, target was found) was present both for the VS condition and for the VS + WM condition. Additional WM load did not affect the VS accuracy for either the single low-salient target condition or the dual-target condition. The WM task accuracy was also similar for the single low-salient target condition and the dual-target condition. This result is inconsistent with the predictions made by the resource depletion theory, which considers object WM as the resource. If the dual-target VS and the color memorization task required the same resources, interference would be observed, but no interference was observed in our experiment.

The RT of the first mouse click was lower for the dual-target condition compared to the single low-salient target condition. As the first target found in the dual-target condition was considered high-salient, this result is quite obvious: it takes less time to find a high-salient target than a low-salient target. There is a slight difference in the VS task and the VS + WM task for the dual-target condition, revealed by rmANOVA but not revealed by pairwise comparisons, supposing a longer RT for the VS + WM condition. This might be explained by the fact that the additional memory task requires more resources and thus extends the search time; this pattern is observed only for high-salient targets (as in the dual-target condition, the first target found is high-salient). A possible explanation may be the floor effect for a single low-salient target: it takes such a long time (4483.23 ms) to find the target and to make a mouse click in the VS condition that the additional memory load does not matter much. However, as the difference between the VS and the VS + WM conditions is not revealed by pairwise comparisons, this difference should be treated with caution.

The RT of the second mouse click (which was made on the low-salient target in the dual-target condition and on the OK button in the single low-salient target condition) was also shorter for the dual-target condition as compared to the single low-salient target condition. This result is consistent with our recent results on dual-target VS where a similar paradigm was used (Gorbunova, 2017) and with the results from a VS task with one target, where the RT increased in trials when the target was absent compared to trials when the target was present (e.g., Moraglia, 1989; Kwak et al., 1991).

Overall, the results of this experiment contradict the idea that object WM depletion is the reason for the SSM effect. Nevertheless, there might be a possibility for separate memory stores for individual, basic features of an object, such as size, color, and orientation (Alvarez and Cavanagh, 2004). In our experiment, the participants are required to search for the targets defined by shape feature – as the rectangles with the same orientation have different positions of the gap, and are perceived as different shapes. At the same time, in the WM task the participants have to memorize the color – another basic feature. For that reason, a color WM task would not affect a shape dual-target VS, whereas shape WM task would. In Experiment 2, we address this issue.

## Experiment 2

In this experiment, we changed the WM task paradigm. We considered that a shape memorization task would be more appropriate to reveal the role of object WM in a dual-target VS with targets defined by shape.

### Materials and Methods

#### Participants

24 new volunteers, 3 male, and 21 female, students of National Research University Higher School of Economics participated in the study. All of them were native Russian speakers with normal or corrected to normal vision. The age varied between 17 and 20 years (*M* = 19.00, *SD* = 0.90). All participants were naive to the experimental hypothesis.

The experiment included three conditions: a WM task, a VS task and a combined task for working memory and visual search (VS + WM). The order of presentation was counterbalanced across subjects. Articulatory suppression was used during the whole experiment to avoid the possibility of verbal coding.

#### Apparatus

The apparatus was the same as used in Experiment 1.

### Working Memory Task

#### Stimuli

**FIGURE 7:**
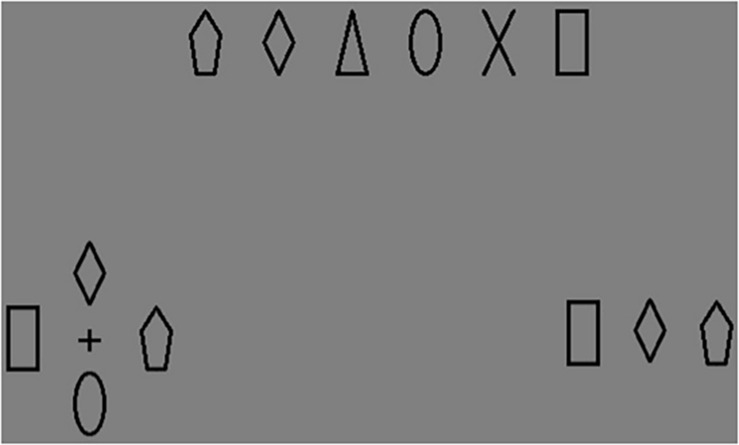
The stimuli used in Experiments 2 and 3 (at the top) and the example of the WM task display of Experiment 2 (at the bottom, on the left), Experiment 3 (at the bottom, on the right).

The stimuli had six varying shapes: pentagon, diamond, triangle, oval, cross, and square. They were drawn with unfilled black lines. The stimuli size was 1.15° × 2.32°. The stimuli and an example of a WM task display are presented in Figure 7. The stimuli were presented on gray background (CIE *xy* = 0.273, 0.304; luminance = 40.897 cd/m^2^). There were always four items per display.

#### Procedure

The design was similar to Experiment 1, except the task of the participants was to memorize the shapes of the stimuli rather than the color. The participant’s task was to remember the initial shapes of the figures of the sample array and to report if the test array is the same as the sample array or not. In 50% trials, the sample array was the same as the test array, in the other 50% trials, one of the shapes was changed.

### Visual Search Task

The stimuli and the procedure were the same as in Experiment 1.

### Visual Search + Working Memory Task

The stimuli and the procedure were similar to Experiment 1, except the task of participant was to memorize the shapes of stimuli rather than the color (as in the WM task from this experiment).

### Results

The apparatus and methods of data analysis were the same as in Experiment 1. Detailed results are presented in Table 2.

**TABLE 2:** The results of Experiment 2.

**Task**	**Condition**	**Index**	**Mean**	**SD**
WM alone task	WM alone	WM accuracy	71.25	7.461495
VS+WM task	WM+VS, no targets	WM accuracy	61.14583	12.06818
	WM+VS, 1 high salient target	WM accuracy	65.83333	14.66411
	WM+VS, 1 low salient target	WM accuracy	67.29167	12.15651
	WM+VS, 2 targets	WM accuracy	61.04167	10.95734
VS alone task	VS alone, no targets	VS accuracy	89.47917	10.63166
	VS alone, 1 high salient target	VS accuracy	71.97917	14.72537
	VS alone, 1 low salient target	VS accuracy	73.95833	16.23397
	VS alone, 2 targets	VS accuracy	64.9556	18.12575
VS+WM task	VS+WM, no targets	VS accuracy	85.59743	10.1687
	VS+WM, 1 high salient target	VS accuracy	67.88471	23.20995
	VS+WM, 1 low salient target	VS accuracy	67.83489	22.60844
	VS+WM, 2 targets	VS accuracy	56.49726	23.45048
VS alone task	VS alone, no targets	1^*st*^ click RT	6327.325	1114.74
	VS alone, 1 high salient target	1^*st*^ click RT	4203.567	587.7411
	VS alone, 1 low salient target	1^*st*^ click RT	4410.755	595.8506
	VS alone, 2 targets	1^*st*^ click RT	3568.186	614.8761
VS+WM task	VS+WM, no targets	1^*st*^ click RT	5869.324	1229.589
	VS+WM, 1 high salient target	1^*st*^ click RT	3741.486	996.5714
	VS+WM, 1 low salient target	1^*st*^ click RT	3832.158	860.5495
	VS+WM, 2 targets	1^*st*^ click RT	3342.801	955.3507
VS alone task	VS alone, no targets	2^*nd*^ click RT	252.8139	105.7471
	VS alone, 1 high salient target	2^*nd*^ click RT	2456.233	801.5538
	VS alone, 1 low salient target	2^*nd*^ click RT	2377.864	750.0756
	VS alone, 2 targets	2^*nd*^ click RT	1711.848	527.4701
VS+WM task	VS+WM, no targets	2^*nd*^ click RT	291.3616	154.9606
	VS+WM, 1 high salient target	2^*nd*^ click RT	2451.511	798.2391
	VS+WM, 1 low salient target	2^*nd*^ click RT	2439.095	962.506
	VS+WM, 2 targets	2^*nd*^ click RT	1761.982	646.4998

### Visual Search

#### Accuracy

**FIGURE 8:**
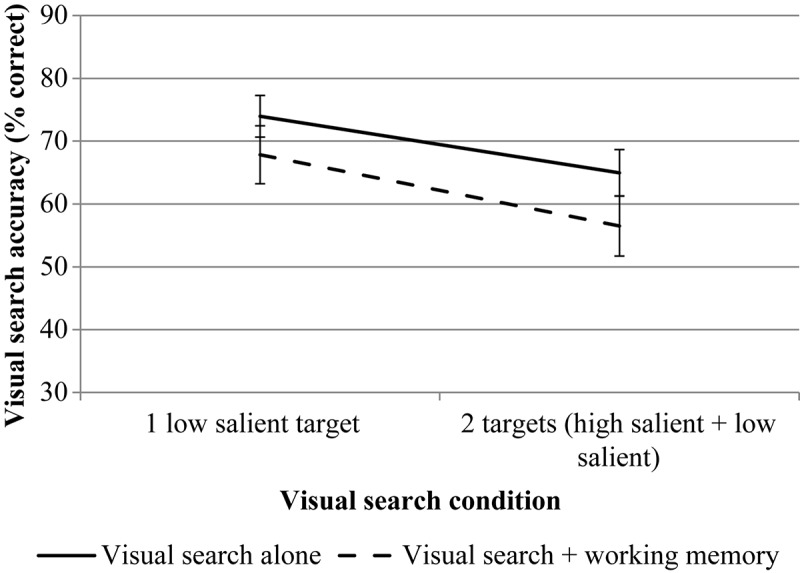
The results of experiment 2 (accuracy data for VS). Error bars represent standard error means.

RmANOVA revealed a significant effect for the number of targets, *F*(1, 23) = 15.70, *p* = 0.001, η*p*^2^ = 0.406. The effect of the WM load is also significant, *F*(1, 23) = 8.01, *p* = 0.009, η*p*^2^ = 0.258. The interaction is not significant, *F*(1, 23) = 0.23, *p* = 0.638, η*p*^2^ = 0.010. The results are presented in Figure 8.

#### Reaction Time

For the first mouse click, rmANOVA revealed a significant effect for the number of targets, *F*(1, 21^5^) = 61.69, *p* = 0.000, η*p*^2^ = 0.746. The effect of the WM load is significant, *F*(1, 21) = 6.08, *p* = 0.022, η*p*^2^ = 0.224. The interaction is not significant, *F*(1, 21) = 2.72, *p* = 0.114, η*p*^2^ = 0.115.

For the second mouse click, rmANOVA revealed a significant effect for the number of targets, *F*(1, 22) = 40.08, *p* = 0.000, η*p*^2^ = 0.646. The effect of the WM load is not significant, *F*(1, 22) = 0.27, *p* = 0.611, η*p*^2^ = 0.012. The interaction is not significant, *F*(1, 22) = 0.15, *p* = 0.705, η*p*^2^ = 0.007.

**FIGURE 9:**
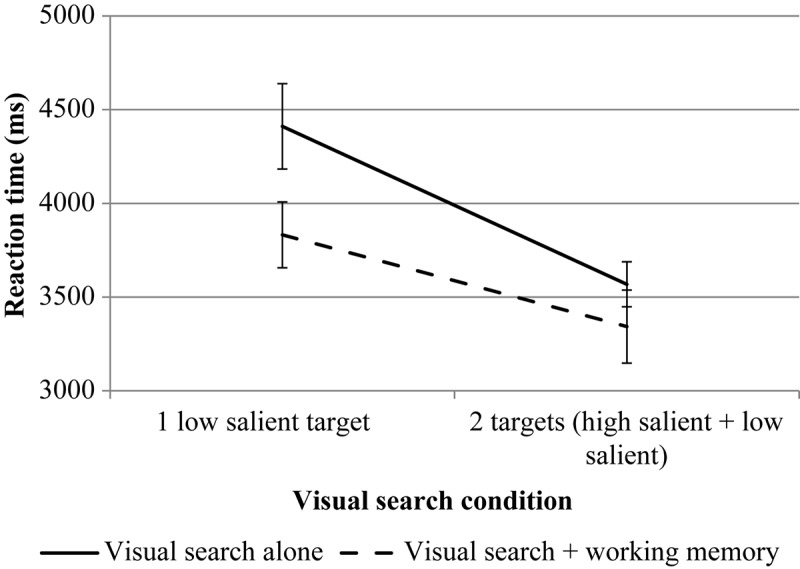
The results of experiment 2 (RT of the first mouse click for VS). Error bars represent standard error means.

**FIGURE 10:**
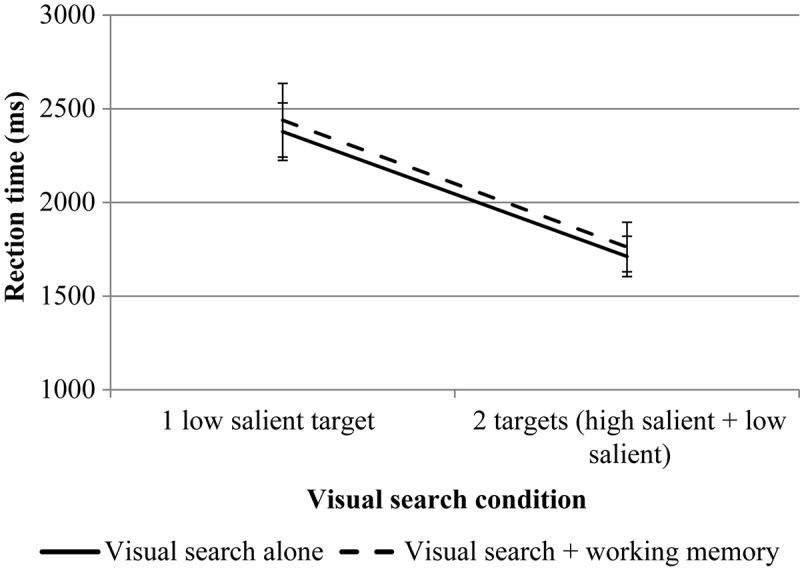
The results of experiment 2 (RT of the second mouse click for VS). Error bars represent standard error means.

The results are presented in Figures 9, 10.

### Working Memory

**FIGURE 11:**
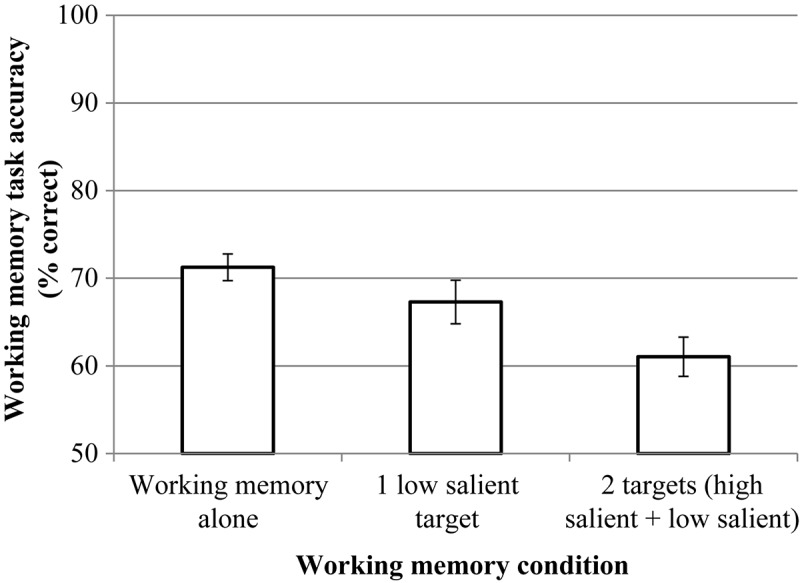
The results of experiment 2 (WM task). Error bars represent standard error means.

RmANOVA revealed a significant effect condition, *F*(2, 46) = 10.86, *p* = 0.002, η*p*^2^ = 0.321. Pairwise comparisons (Bonferroni corrected) revealed significant differences between the dual-target condition (*M* = 61.04, *SD* = 10.96) and the single low-salient target condition (*M* = 67.29, *SD* = 12.16), *p* = 0.008^6^ and between the dual-target condition (*M* = 61.04, *SD* = 10.96) and the WM condition (*M* = 71.25, *SD* = 7.46), *p* = 0.000. The differences between the WM condition (*M* = 71.25, *SD* = 7.46) and the single low-salient target condition (*M* = 67.29, *SD* = 12.16) are not significant, *p* = 0.105. The results are presented in Figure 11.

### Discussion

As in Experiment 1, this experiment revealed a significant effect for the number of targets: SSM was present both for the VS condition and for the VS + WM condition. The role of the WM load was significant: the accuracy decreased in the VS + WM condition compared to the VS condition both for the single and the dual-target conditions. Nevertheless, the accuracy decreased with the additional WM load equally for the single and the dual-target conditions. This may indicate the general interference between the WM and the VS tasks, but no specific deterioration in the dual-target search condition. However, this specific interference is revealed in the WM task: the accuracy for the WM task decreased for the dual-target condition compared to the WM and the VS + WM single-target condition.

These results indicate that the dual-target search and the shape memorization task share a common resource. However, this is not a clear argument for the object memory resource depletion theory, assuming that the representation of the first target is loaded in WM system, depleting its resources and causing the second target omission. The additional WM task had no specific influence on the dual-target search, but the additional VS task decreased the WM task accuracy. This could mean that the representations of the first and the second target are loaded in WM system and inhibit WM recall of the previously encoded stimuli. The single-target trials do not cause this violation because the WM capacity is big enough to hold the representations of shapes for memorization and the first target identity, whereas the second target representation causes WM overload and the decrease in the accuracy of WM recall. The second target omission is not related to WM resource depletion, at least in a direct way.

The number of items on the display was equal for single and dual target trials, and the overall search time was reduced for dual-target trials as compared to single-target trials (that is, more items in the display are checked to see whether they have the target feature), but the memory task performance was reduced for the dual-target trials, but not for single-target trials. Assuming that all scanned items (both targets and distracters) are processed in WM, this might be due to the target occupying a different position in VWM than each of the candidate items. Previous research revealed that found targets may have a privileged representation in memory as compared to distracters, for example, the visual details of search targets are remembered better as compared to distracter objects unrelated to the search target (Williams et al., 2005). To this end, one can expect found targets to consume more WM resources as compared to distracters.

The RT for the first mouse click is surprisingly lower for the VS + WM condition compared to the VS condition. This pattern is similar for both the single- and the dual-target condition. This might reflect the tendency of participants to make a mouse click as fast as possible in the VS + WM condition in order not to lose the items held in WM during the VS trial, as well as a speed-accuracy trade off.

The RT of the first mouse click was lower for the dual-target condition compared to the single low-salient target condition. The RT of the second mouse click was also lower for the dual-target condition compared to the single low-salient target condition. These results are the same as the findings of Experiment 1.

Overall, the results of this experiment revealed interference between the WM and the VS tasks. Yet, the overall pattern of results shows a speed-accuracy trade off: the VS condition had better accuracy and a faster first mouse click compared to the combined condition. Another point is the lower WM alone accuracy in the WM alone condition compared to Experiment 1, and greater task complexity as reported by the subjects. For that reason, another experiment was conducted.

## Experiment 3

The design of this experiment was similar to Experiment 2. The only difference is that the WM task included three objects, instead of four, for memorization. This manipulation was conducted in order to reduce overall task complexity.

### Materials and Methods

#### Participants

24 new volunteers, 5 male, and 19 female, students of National Research University Higher School of Economics participated in the study. All of them were native Russian speakers with normal or corrected to normal vision. The age varied between 19 and 22 years (*M* = 20.17, *SD* = 0.76). All participants were naive to the experimental hypothesis.

The experiment included three conditions: a WM task, a VS task and a combined task for working memory and visual search (VS + WM). The order of presentation was counterbalanced across subjects. Articulatory suppression was used during the whole experiment to avoid the possibility of verbal coding.

#### Apparatus, Stimuli, and Procedure

The apparatus, stimuli and procedure were the same as used in Experiments 1 and 2, except on each trial three (instead of four) shapes were displayed – both in WM alone condition and in VS+WM condition. The stimuli and an example of the WM task display are presented in Figure 7.

### Results

The apparatus and methods of data analysis were the same used in Experiments 1 and 2. Detailed results are presented in Table 3.

**TABLE 3:** The results of Experiment 3.

**Task**	**Condition**	**Index**	**Mean**	**SD**
WM alone task	WM alone	WM accuracy	85.79167	9.541576
VS+WM task	WM+VS, no targets	WM accuracy	78.54167	13.24757
	WM+VS, 1 high salient target	WM accuracy	81.5625	15.05086
	WM+VS, 1 low salient target	WM accuracy	79.375	13.63838
	WM+VS, 2 targets	WM accuracy	75.20833	15.61975
VS alone task	VS alone, no targets	VS accuracy	90.625	8.573531
	VS alone, 1 high salient target	VS accuracy	76.25	14.81773
	VS alone, 1 low salient target	VS accuracy	73.75	18.38596
	VS alone, 2 targets	VS accuracy	65.65467	21.67495
VS+WM task	VS+WM, no targets	VS accuracy	78.72762	17.56911
	VS+WM, 1 high salient target	VS accuracy	74.40494	13.84429
	VS+WM, 1 low salient target	VS accuracy	72.53367	22.44321
	VS+WM, 2 targets	VS accuracy	67.6629	21.71855
VS alone task	VS alone, no targets	1^*st*^ click RT	6389.581	1159.015
	VS alone, 1 high salient target	1^*st*^ click RT	4419.412	630.7079
	VS alone, 1 low salient target	1^*st*^ click RT	4699.97	710.591
	VS alone, 2 targets	1^*st*^ click RT	3860.571	667.3049
VS+WM task	VS+WM, no targets	1^*st*^ click RT	6448.968	1168.982
	VS+WM, 1 high salient target	1^*st*^ click RT	4387.281	890.1741
	VS+WM, 1 low salient target	1^*st*^ click RT	4536.122	850.2184
	VS+WM, 2 targets	1^*st*^ click RT	4084.631	1206.384
VS alone task	VS alone, no targets	2^*nd*^ click RT	286.0606	167.6711
	VS alone, 1 high salient target	2^*nd*^ click RT	2518.67	759.3444
	VS alone, 1 low salient target	2^*nd*^ click RT	2491.664	623.4778
	VS alone, 2 targets	2^*nd*^ click RT	2020.995	578.9655
VS+WM task	VS+WM, no targets	2^*nd*^ click RT	400.4899	262.2409
	VS+WM, 1 high salient target	2^*nd*^ click RT	2830.399	1226.713
	VS+WM, 1 low salient target	2^*nd*^ click RT	2911.086	1312.184
	VS+WM, 2 targets	2^*nd*^ click RT	1955.551	533.6348

### Visual Search

#### Accuracy

**FIGURE 12:**
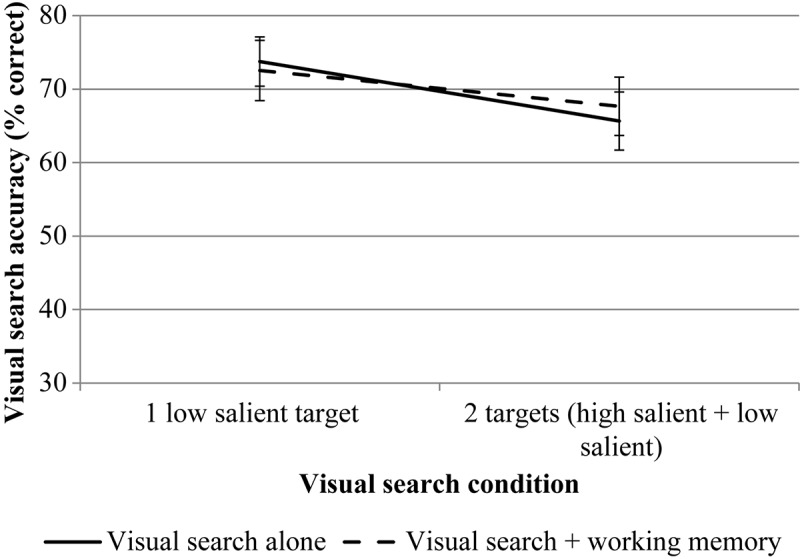
The results of experiment 3 (accuracy data for VS). Error bars represent standard error means.

RmANOVA revealed a significant effect for the number of targets, *F*(1, 23) = 6.96, *p* = 0.015, η*p*^2^ = 0.232. The effect of the WM load is not significant, *F*(1, 23) = 0.01, *p* = 0.910, η*p*^2^ = 0.001. The interaction is not significant, *F*(1, 23) = 0.62, *p* = 0.441, η*p*^2^ = 0.026. The results are presented in Figure 12.

#### Reaction Time

For the first mouse click, rmANOVA revealed a significant effect for the number of targets, *F*(1, 23) = 33.87, *p* = 0.000, η*p*^2^ = 0.596. The effect of the WM load is not significant, *F*(1, 23) = 0.03, *p* = 0.866, η*p*^2^ = 0.001. The interaction is significant, *F*(1, 23) = 5.29, *p* = 0.031, η*p*^2^ = 0.187. Pairwise comparisons revealed no significant differences between different levels of load (the VS task compared to the VS + WM task) both for the single low-salient target condition, *p* = 0.277 and for the dual-target condition, *p* = 0.347.

For the second mouse click, rmANOVA revealed a significant effect for the number of targets, *F*(1, 23) = 32.46, *p* = 0.000, η*p*^2^ = 0.585. The effect of the WM load is not significant, *F*(1, 23) = 1.35, *p* = 0.257, η*p*^2^ = 0.055. The interaction is significant, *F*(1, 23) = 5.34, *p* = 0.030, η*p*^2^ = 0.188. Pairwise comparisons revealed no significant differences between different levels of load (the VS task compared to the VS + WM task) both for the single low-salient target condition, *p* = 0.080 and for the dual-target condition, *p* = 0.608.

**FIGURE 13:**
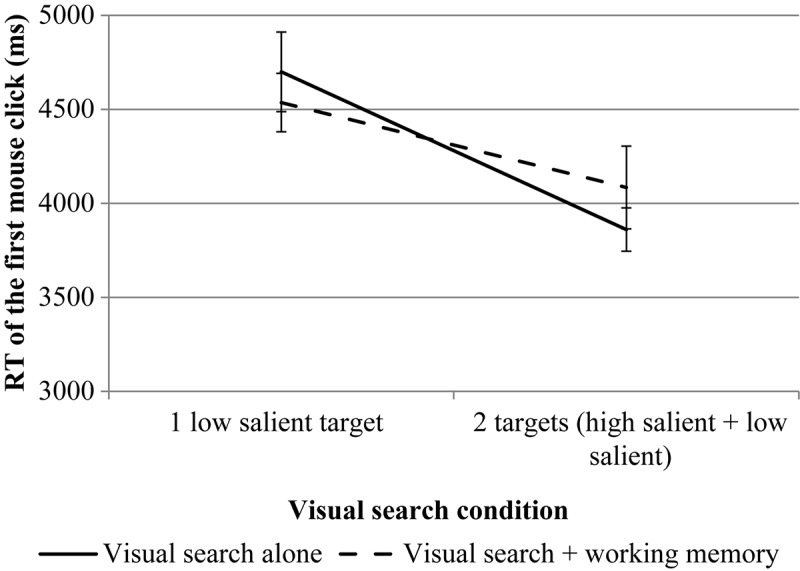
The results of experiment 3 (RT of the first mouse click for VS). Error bars represent standard error means.

**FIGURE 14:**
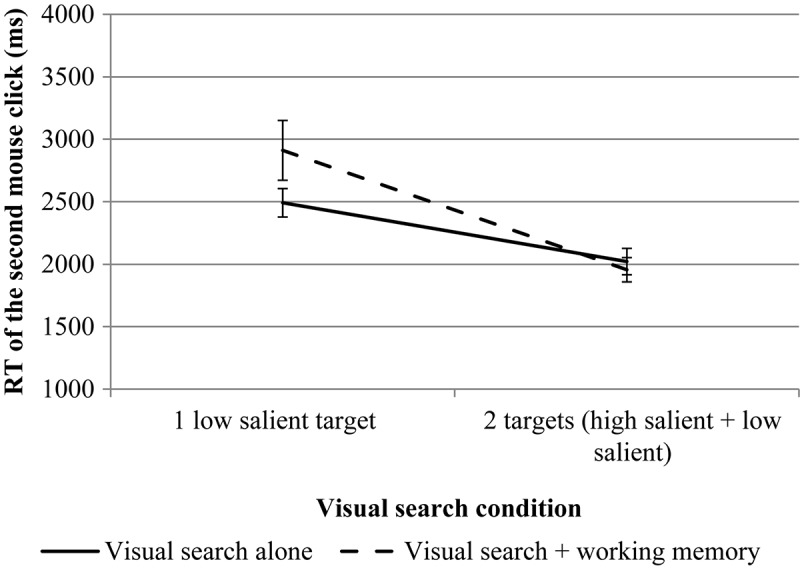
The results of experiment 3 (RT of the second mouse click for VS). Error bars represent standard error means.

The results are presented in Figures 13, 14.

#### Working Memory

**FIGURE 15:**
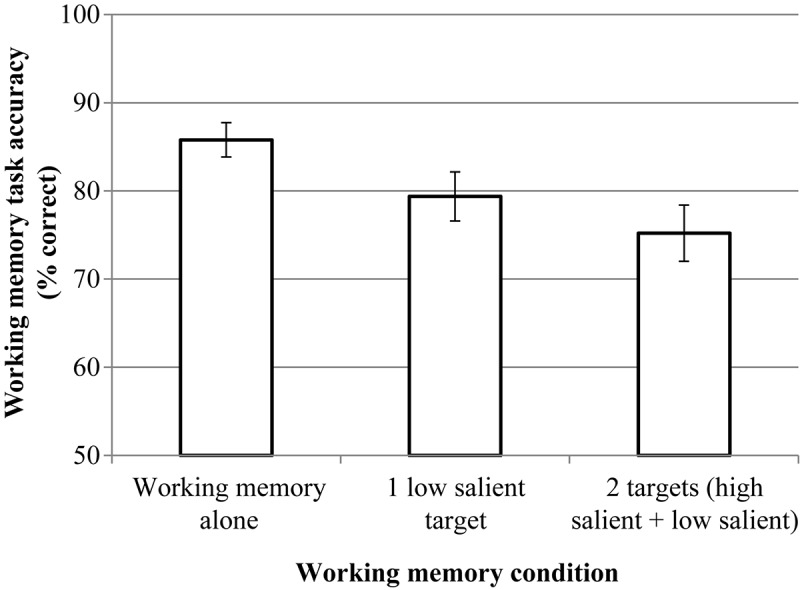
The results of experiment 3 (WM task). Error bars represent standard error means.

RmANOVA revealed a significant effect for the condition, *F*(2, 37) = 14.61, *p* = 0.000, η*p*^2^ = 0.388. Pairwise comparisons (Bonferroni corrected) revealed significant differences between the dual-target condition (*M* = 75.21, *SD* = 15.62) and the single low-salient target condition (*M* = 79.38, *SD* = 13.64), *p* = 0.010^7^ and between the dual-target condition (*M* = 75.21, *SD* = 15.62) and the WM condition (*M* = 85.79, *SD* = 9.54), *p* = 0.000. The differences between the WM condition (*M* = 85.79, *SD* = 9.54) and the single low-salient target condition (*M* = 79.38, *SD* = 13.64) are also significant, *p* = 0.003. The results are presented in Figure 15.

### Discussion

As Experiments 1 and 2, this experiment revealed a significant effect for the number of targets: SSM was present both for the VS condition and for the VS + WM condition. The results of this experiment are the same as for Experiment 2 for the WM task, where the accuracy of the WM task decreased with the number of targets in the VS task, revealing interference between the dual-target search task and the WM task. This is consistent with the idea of interference between the WM and VS tasks. The WM task accuracy is comparable with Experiment 1, indicating similar task complexity.

The results of this experiment differ from Experiment 2 for the VS task: in this experiment, no difference in accuracy was found for the VS condition and the VS+WM condition. The effect of the WM load is also not significant for the first and second mouse clicks. This reflects the absence of a speed-accuracy trade-off, observed in the previous experiment.

In this experiment, we also replicated the results for RT data from Experiments 1 and 2: The RT of the first and the second mouse click was lower for the dual-target condition compared to the single low-salient target condition. The difference between Experiment 1 and Experiment 2 is the interaction between the WM load factor and the number of targets: the VS + WM task assumes a slight increase of the RT for the single low-salient target condition; however, this was not detected by pairwise comparisons. This might be a sign that an increased WM load extends the time required to report the target absence.

One of the possible confounds might be the difference in retention interval for WM alone condition (4000 ms) and VS+WM condition (the response for WM task was given after the VS task response), what could explain the difference in response accuracy in WM task in WM alone and VS+WM condition. However, according to RT data for VS+WM condition the time of retention was larger for single target condition as compared to dual-target condition, whereas the WM response accuracy was less for dual-target condition, so the pattern is opposite. In this way, the interference is increased in dual-target trials as compared to single-target trials, even though single-target trials assume longer retention. While the subject is searching for the second target in single-target trial, the distracters that he is observing seem to be encoded differently as compared to the second target when it is found in dual-target trials. This leads us to idea that targets occupy a different position in VWM than distracters. As we can assume from experiment 1, the nature of WM task matters in that case, as color-memorization WM task performance did not differ for single and dual-target trials. One of the possible explanations might be related to the idea of active and passive WM representations. The target identity is relevant to the attention template, whereas the distracters are not. To this end, storage of template-relevant object representation would cause more interference with the secondary WM task, which is the case here.

Overall, this experiment replicated the results of Experiment 2 in the part related to the shape WM task and the dual-target VS interference, with comparable results to Experiment 1 for the WM task accuracy and without a speed-accuracy trade-off for the VS task, observed in Experiment 2. In this way, the results of this experiment revealed a violation of the WM task with the additional dual-target VS task but not a violation of the dual-target VS task with the additional WM task.

There is a slight difference in WM task results of this experiment from the Experiment 2. In Experiment 2, the additional VS task did not violate WM performance when only one target was displayed, whereas two targets decreased performance in WM task. In Experiment 3, even one target decreased WM performance, and two targets contributed more decrease in performance. In Experiment 2, the accuracy was equal in condition with four WM objects and one VS target (5 objects overall) and WM alone condition (4 objects). In Experiment 3, on the contrary, the accuracy in condition with three WM objects and two VS objects (5 objects overall) was reduced as compared to conditions with three WM objects and one VS object (4 objects overall). In addition, accuracy in the WM alone condition (4 objects) in Experiment 2 was worse as compared to the dual-target VS+WM condition (5 objects) in Experiment 3.

This may be due to the different capacities required for shape memorization (“memory for recall”) and for VS target memorization (“memory for search”). This is evidenced by the differences in the WM alone condition in Experiment 2 and Experiment 3: the performance is reduced in Experiment 2, whereas the only difference relates to the number of objects for memorization. If “memory for recall” requires more capacity, one additional VS target may not affect performance significantly when memory is overloaded with four objects, but additional VS target would affect if there are only three objects in memory. An alternative explanation might be due to artifacts with a speed-accuracy trade-off, as observed in Experiment 2.

## General Discussion

Three experiments were conducted to reveal the role of WM deficit in SSM errors. The first experiment investigated the role of object WM using a classical color change detection task. In the second and the third experiment, a modified change detection task was applied, using shape as the relevant feature. The second and third experiments revealed significant interference between the WM and VS tasks, whereas the first experiment did not reveal this pattern.

A dual-target VS interferes with the object WM task when the features used in the WM task are the same features that define the VS task: the interference is observed for the shape-based WM task and the shape-based VS task, but not for the shape-based VS task and the color-based WM task. This is an argument for separate storage of different features in WM.

Overall, an additional dual-target VS task decreases WM task performance, but an additional WM task does not decrease dual-target VS task performance. This is the argument for WM recall being inhibited by VS stimuli and for the idea of general VS and WM task interference. But this cannot be assumed as an argument for WM resource depletion theory as the second target omission probability does not increase with an additional WM load. Nevertheless, this result might be related to the participant’s strategy to sacrifice their performance in WM task in order to perform the VS task with equal efficiency regardless to the number of targets. However, an additional analysis (see footnote 2) for all trials revealed the results equal to the analysis that was conducted only for the correctly answered WM task trials. A more elaborated point might be obtained through the additional experiment with reward for each correct WM task trial but without any reward for VS task. Moreover, even if SSM errors are not related to WM deficit, they might be relevant to attentional resource depletion.

Although our experiments were conducted in the frame of WM resource depletion theory as the explanation of SSM errors, the relation to perceptual similarity theory should be discussed as well. According to that theory, the first-found target creates a representation [similar to “an attentional template” (e.g., Carlisle et al., 2011)], which is responsible for creating a perceptual bias. The subject tends to search for perceptually similar targets and to miss perceptually dissimilar targets. This explanation is not necessarily contradictory to the resource depletion account: an attentional template can both cause resource overload and create perceptual bias. After the first-found target is encoded in WM, an attentional template is created. This attentional template might be stored in WM and guide the subsequent VS. From this point of view, it is still possible to discuss the role of WM as the relevant explanation of SSM errors, but not from the point of resource depletion.

Another result of our experiments is the difference in additional memorized shape cost and additional VS target cost: additional memorized shape affected performance more as compared to an additional VS target. This may refer to the difference in capacity required for shape memorization (“memory for recall”) and for observed targets’ identities (“memory for search”).

Overall, the results of our study revealed no effect of an additional WM task on second target detection in dual-target VS. To this end, SSM errors are not related to WM resource depletion. On the contrary, WM task performance was violated by dual-target VS as compared to single-target VS. We assume that the target representations are loaded to WM and inhibit the recall of the previously encoded stimuli, when they share the same feature to be recalled.

Future experiments might be related to reveal the role of WM load in light of the perceptual similarity account. It may include the manipulation of both WM load and the perceptual similarity in dual-target VS. Another manipulation could use different stimuli for the WM task, more like the VS task stimuli.

## Data Availability

The datasets generated for this study are available on request to the corresponding author.

## Ethics Statement

All experiments reported in this manuscript were carried out in accordance with the Declaration of Helsinki and the existing Russian and international regulations concerning ethics in research. All participants provided written informed consent. We did not seek approval by an institutional review board for the experiments because it is not required for a study of the type reported in this manuscript.

## Author Contributions

EG conceptualized the study, responsible for experimental planning, performed programming, carried out data analysis, and prepared the manuscript. KK, SL, and IM were responsible for data collection and analysis, and manuscript preparation.

## Conflict of Interest Statement

The authors declare that the research was conducted in the absence of any commercial or financial relationships that could be construed as a potential conflict of interest.

## Footnotes

1 Experiment data are available as additional file. PsychoPy files are available by the request to other researchers for purposes of replicating the procedure or reproducing the results.

2 The analysis for all trials was conducted as well, the results are the same.

3 The *df* for RT data is less than 29 because some of the participants failed to find any targets in some conditions, that’s why the dataset for RT contained less measurements.

4 The Bonferroni corrected alpha is 0.017 due to three comparisons conducted.

5 The *df* for RT data is less than 23 because some of the participants failed to find any targets in some conditions, that’s why the dataset for RT contained less measurements.

6 The Bonferroni corrected alpha is 0.017 due to three comparisons conducted.

7 The Bonferroni corrected alpha is 0.017 due to three comparisons conducted.
